# 2066. Synergistic Bacteriophage-Antibiotic Combinations Against High Inoculum DNS MRSA

**DOI:** 10.1093/ofid/ofad500.136

**Published:** 2023-11-27

**Authors:** Ashlan J Kunz Coyne, Kyle Stamper, Callan Bleick, Arnold S Bayer, Susan Lehman, Michael J Rybak

**Affiliations:** University of Kentucky, Detroit, Michigan; Wayne State University, Detroit, Michigan; Anti-Infective Research Laboratory, College of Pharmacy and Health Sciences, Wayne State University, Detroit, MI, Wayne State University School of Medicine, Department of Microbiology and Immunology, Detroit, MI, Detroit, Michigan; Lundquist Institute-Harbor UCLA Medical Center, Torrance, California; Center for Biologics Evaluation and Research, US Food and Drug Administration, Silver Spring, Maryland; Eugene Applebaum College of Pharmacy and Health Sciences, Detroit, Michigan

## Abstract

**Background:**

Phage-antibiotic combinations (PAC) have been proposed for high inoculum daptomycin-nonsusceptible (DNS) MRSA infections refractory to conventional therapy. We studied PAC with synergistic activity against two DNS MRSA clinical bloods isolates (C4 and C37; DAP MIC = 4 ug/mL).

**Methods:**

PAC containing DAP and/or ceftaroline (CPT) (each at ½ MIC) plus a 2-phage cocktail (Intesti13 and Sb-1, at a range of multiplicities of infection [MOI]) were tested at a high MRSA inoculum (10^9^ CFU/mL) using: **i**) modified checkerboard (CB) minimum inhibitory concentration (MIC); and **ii**) 24h time-kill assays (TKA). Synergistic activity in CB assays was defined as either: a fractional inhibitory concentration (FIC) index ≤ 0.5 in modified CB assays; or a ≥ 2 log_10_ CFU/mL reduction by PACs vs the most active single-agent regimen. Significant differences between regimens were assessed by ANOVA with Tukey’s *post hoc* modification (*P* < 0.05).

**Results:**

By CB assay, synergistic activity was demonstrated with Intesti13 + Sb1 (MOI of 10 to 0.01) plus either DAP or DAP + CPT (FIC ≤ 0.5 for each combination). In 24h TKA vs C4, Intest13 + Sb1 (MOI 1 and 0.1) plus either DAP or DAP + CPT demonstrated robust synergistic activity (-Δ7.21 and -Δ7.39 log_10_ CFU/mL, respectively) vs. the next most effective regimen of CPT + Intesti13+Sb1 (*P* < 0.05 each). Against C37, Intesti13 + Sb1 (MOI of 1 and 0.1) with CPT or DAP + CPT were equally potent and effective regimens (-Δ7.14 log_10_ CFU/mL each), but neither were significantly better than the synergistic regimen of DAP + Intesti13 + Sb1 (-Δ6.65 log_10_ CFU/mL).

**Figure 1.**
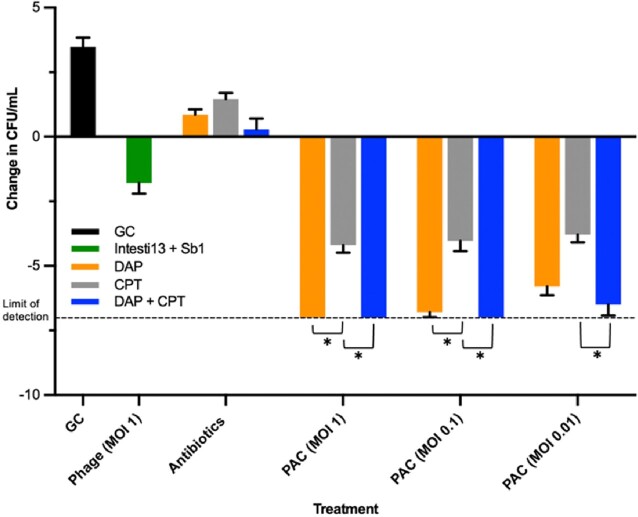
Bacterial quantification in 24 h high inoculum (10^9 CFU/mL) TKA of DAP and CPT combined with phages Intesti13 and Sb1 against DNS MRSA strain C4.

These studies demonstrated positive phage-antibiotic synergy (PAS), with phage cocktail Intesti13 + Sb1 and either DAP or DAP + CPT. Addition of these phages to DAP or DAP + CPT combinations caused bactericidal and synergistic killing vs. CPT + phages. *, P<0.05. Abbreviations: CFU: colony forming units, GC: growth control, DAP: daptomycin, CPT: ceftaroline, MOI: multiplicity of infection, PAC: phage-antibiotic combination.

**Figure 2.**
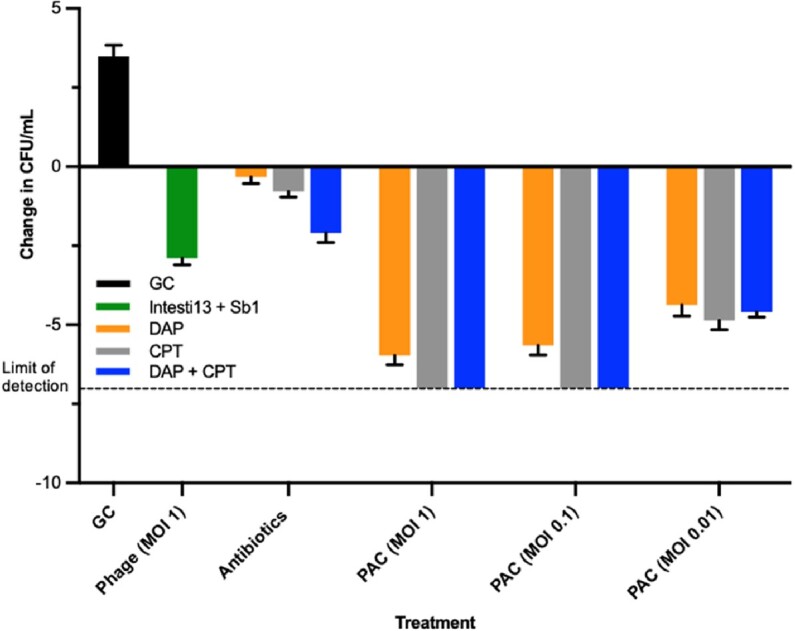
Bacterial quantification in 24 h high inoculum (10^9 CFU/mL) TKA of DAP and CPT combined with phages Intesti13 and Sb1 against DNS MRSA strain C37.

These studies demonstrate positive phage-antibiotic synergy (PAS) with phage cocktail Intesti13 + Sb1 added to DAP, CPT, and DAP + CPT at an MOI of 1 and 0.1. Abbreviations: CFU: colony forming units, GC: growth control, DAP: daptomycin, CPT: ceftaroline, MOI: multiplicity of infection, PAC: phage-antibiotic combination.

**Conclusion:**

The two-phage cocktail used (Intesti13 + Sb1) demonstrated impressive synergistic activity against two DNS MRSA isolates in combination with DAP or DAP + CPT. Further experimental *in vivo* investigations of these candidate PACs, for treatment of high inoculum DNS MRSA infections (e.g., infective endocarditis) is warranted.

**Disclosures:**

**Arnold S. Bayer, MD**, Akagera Medicines: Grant/Research Support|ContraFect Corporation: Grant/Research Support **Michael J. Rybak, PharmD, PhD, MPH**, Abbvie, Merck, Paratek, Shionogi, Entasis, La Jolla, T2 Biosystems: Advisor/Consultant

